# The Internet Hospital as a Telehealth Model in China: Systematic Search and Content Analysis

**DOI:** 10.2196/17995

**Published:** 2020-07-29

**Authors:** Yangyang Han, Reidar K Lie, Rui Guo

**Affiliations:** 1 School of Public Health Capital Medical University Beijing China; 2 Department of Philosophy University of Bergen Bergen Norway

**Keywords:** Internet hospital, telehealth, telemedicine, ehealth, digital health, digital medicine, health services research, China

## Abstract

**Background:**

The internet hospital is an innovative organizational form and service mode under the tide of internet plus in the Chinese medical industry. It is the product of the interaction between consumer health needs and supply-side reform. However, there has still been no systematic summary of its establishment and definition, nor has there been an analysis of its service content.

**Objective:**

The primary purpose of this study was to understand the definition, establishment, and development status of internet hospitals.

**Methods:**

Data on internet hospitals were obtained via the Baidu search engine for results up until January 1, 2019. Based on the results of the search, we obtained more detailed information from the official websites and apps of 130 online hospitals and formed a database for descriptive analysis.

**Results:**

By January 2019, the number of registered internet hospitals had expanded to approximately 130 in 25 provinces, accounting for 73.5% of all provinces or province-level municipalities in China. Internet hospitals, as a new telehealth model, are distinct but overlap with online health, telemedicine, and mobile medical. They offer four kinds of services—convenience services, online medical services, telemedicine, and related industries. In general, there is an underlying common treatment flowchart of care in ordinary and internet hospitals. There are three different sponsors—government-led integration, hospital-led, and enterprise-led internet hospitals—for which stakeholders have different supporting content and responsibilities.

**Conclusions:**

Internet hospitals are booming in China, and it is the joint effort of the government and the market to alleviate the coexistence of shortages of medical resources and wasted medical supplies. The origin of internet hospitals in the eastern and western regions, the purpose of the establishment initiator, and the content of online and offline services are different. Only further standardized management and reasonable industry freedom can realize the original intention of the internet hospital of meeting various health needs.

## Introduction

During the past five years, China has been committed to promoting the use of the internet to provide medical services, although it is still in the exploratory stage [1]. Internet connections, in general, have brought about a significant change to the Chinese economy, and there is no doubt that it can improve the quality of service and provide convenience for people. As a supplement to offline health services, online health services are of great significance in alleviating the relative shortage of medical resources in China [2]. With the development of technology, online health service levels have continually improved, which has led traditional medical resources to gradually become networked.

China has 904 million internet users, with an internet penetration rate of 64.5%, according to the China Internet Network Information Center in April 2020 [3]. The vast number of internet users constitute China's booming consumer market. Mobile payment is becoming more and more popular in daily life, and user habits of paying for online medical services based on knowledge are also becoming established [4]. From April 2018 to April 2019, China's internet medical users grew from 28 million to 45 million, a year-on-year increase of 59.9% [5]. Ping An Good Doctor, one of the representative internet hospitals, had 300 million registered users as of October 2019, equivalent to 1 in every 3 Chinese netizens having used it [6], and the number of visitors exceeded 1.11 billion from January to April 2020 during the COVID-19 pandemic [7]. The age range of internet hospital users is wide, and the number of middle-aged and elderly users is increasing [8].

*Internet hospitals* is a term with a somewhat vague meaning. In general, it is an internet medical platform combining online and offline access for medical institutions to provide a variety of telehealth services directly to patients. It extends medical resources from the hospital to the internet by using information technology, and it develops online medical services and health services [9]. It can be as simple as using the internet to make and manage appointments or renew prescriptions; however, it can also be a means for Chinese citizens to communicate face-to-face with doctors via internet pages or public therapeutic platforms in apps such as WeChat (mobile social media apps) [10]. Internet hospitals are online platforms but need to be based in existing institutions with consistent online and offline service supervision. They include both medical and nonclinical services but can only use internet technology to provide safe and appropriate medical services for the follow-up of common and chronic diseases online [11]. Doctors are allowed to prescribe treatment for related diseases online after accessing the patient's medical records [11].

Telehealth is a medical service model similar to internet hospitals in China and is used in settings throughout the world [12,13]. It commonly describes a wide range of diagnostic and management modalities, education, and other related activities within health care [14]. It is expanding beyond telemedicine to cover nonclinical events such as appointment scheduling, continuing medical education, and physician training [14].

In the United States, telehealth or telemedicine has been defined in all states, and some of these services by video can be reimbursed [15,16]. Many states consider online services inadequate for building doctor-patient relationships and prescribing treatments, so some allow online diagnosis and prescription of drugs while others restrict them [15]. A growing number of states are passing legislation to guide health care professional boards to adopt standards of practice for providers using telehealth [15]. One US telehealth platform, Amwell, an internet-based health care giant, has more than 150 million insured users, serving over 55 health plans, more than 240 medical groups, and more than 2,000 hospitals [17]. In terms of strategy and governance, studies have shown that only 2 countries in the European Union are in the management stage of policy supervision, and 16 countries are considered to be in the initial and temporary stage, meaning that most countries in the EU have no formal definition of telemedicine services or only small short-term and independent pilot developments [18]. Meanwhile, in Japan, access to telehealth clinical functions was restricted to health consultations, and only during the COVID-19 pandemic did the government allow patients to receive medical care and receive prescription via the internet rather than by going to a physical hospital [19,20].

However, in China, the current situation developed after an initial period of growth in internet hospital services when Chinese health authorities ordered a temporary halt in the development in order to develop a more appropriate set of regulations [21]. The first internet hospital in China was established in Guangdong Province in 2014 [22]. Then, China began to promote this new model of online health care to develop internet health services actively, to provide convenience services through the mobile internet, to encourage internet enterprises to cooperate with medical institutions, and to establish a medical network information platform. Thus, internet hospitals began to appear in some areas, gradually. In 2016, the First Affiliated Hospital of Zhejiang University established Zhejiang First internet hospital and was headed by the tertiary hospital [23]. However, by 2017, the government re-examined and approved the abolition of internet hospitals, cloud hospitals, and network hospitals [21]. By 2018, the state again encouraged medical institutions to use information technology such as the internet to expand the space and content of medical services and to build a comprehensive online and offline medical service model covering prognosis, diagnosis, and postdiagnosis [11]. Medical institutions were again allowed to develop internet hospitals and to provide safe and appropriate medical services using internet technology [11]. In 2018, the government formulated specific regulations on internet hospital management, including diagnosis and treatment, telemedicine, and other online aspects. This marked the beginning of the standardized development of internet hospitals [11].

Three documents were issued on July 17, 2018 by the National Health Commission of the People’s Republic of China and National Administration of Traditional Chinese Medicine: Internet Diagnosis and Treatment Management Measures (trial implementation), Internet Hospital Management Measures (trial implementation), and Telemedicine Service Management Standards (trial implementation) [11]. It was the first time that China put forward detailed regulations on internet hospitals, signifying that internet hospitals had entered the stage of standardized development [11]. Chinese internet hospitals have experienced ups and downs, after twists and turns of the embryonic stage, and are now entering the rapid development stage of standardization. 

In order to capture the current state of development of internet hospitals in China, we carried out a study covering internet hospitals (up to the beginning of 2019) to analyze the construction mode and content of internet hospitals and provide corresponding suggestions for construction. 

## Methods

This study obtained data on internet hospitals through the Baidu search engine and Apple and Android app stores, manually. Baidu's dominant role in internet searching in China is comparable to the role of Google in Western countries, on both desktop and mobile devices, with approximately 70% to 80% market share. People can search for queries by entering keywords.

First, we searched for the official internet hospital name from April 6, 2019 to April 12, 2019. Before searching in Baidu, we sorted and analyzed policies of internet hospitals at the national level on the Website of the State Council. Internet Hospital Management Regulations stressed that internet hospitals should use the name of the hospital with the addition of the term *internet hospital* [11]. Some hospitals use the name of *cloud hospital* or *web hospital* as an auxiliary reference. So, we searched according to the internet hospital naming rules, following the Baidu Robots Protocol. Our inclusion criteria were internet hospitals established before January 1, 2019, and we included direct and indirect search results (direct: web items; indirect: government announcements and page content under web item). This resulted in a list of 130 internet hospital names in our Excel (Microsoft Inc) database.

Second, we queried the relevant information about the internet hospital name in Baidu, inquired from the internet hospital official website, and downloaded the internet hospital app from the app store to obtain the following variables to build a database used in the statistical analysis: establishment time (precise to the month), establishment location, service content provided by technical cooperation parties, and media (pages, public medical platforms, or apps) in contact with people. The study focused on the overall development of the internet hospital industry, so we did not collect the number of patients and doctors in each hospital.

Finally, the data obtained from the 130 internet hospitals were entered into the Excel database and analyzed.

## Results

### The Structure of Internet Hospitals

Our study of the available information about internet hospitals revealed that the term is used for a number of different types of activities. Telemedicine was launched first, and then mobile medicine and online medicine became popular. Generally speaking, online medicine, mobile medicine, and telemedicine are all covered by the concept of internet hospital. In Table 1, we have summarized the relationships between the various uses of the term internet hospital.

**Figure 1:**
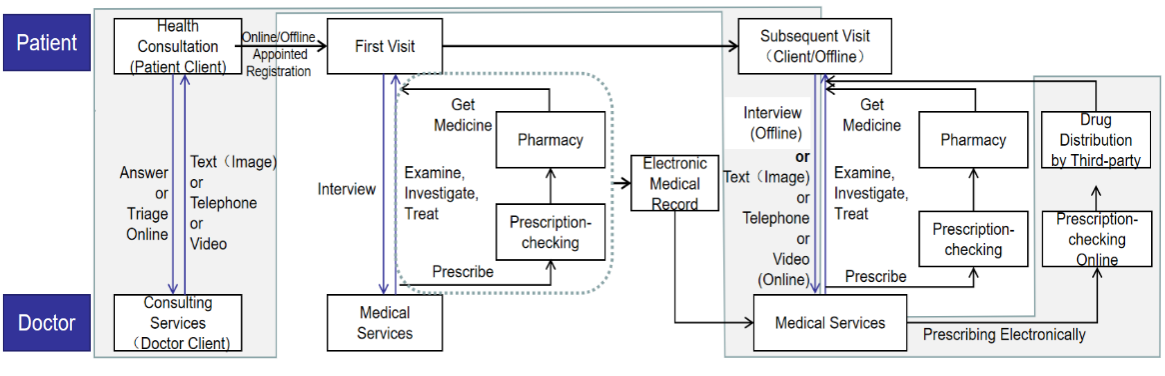
Hospital treatment flowchart.

Based on the data we obtained, we constructed a flowchart between offline hospitals and online hospitals by summarizing the treatment process of existing internet hospitals based on doctor-patient interaction (Figure 1). Patients can consult online before going to the offline hospital to collect relevant professional health information and understand their current situation. If they need to go to a hospital, there can be a more extensive and reliable consultation that can assist the hospital in triaging. Next, patients can choose to register online or go to a hospital for a preliminary examination. After the first visit, patients can choose between online or offline ways to check the disease prognosis, treatment effects, and further treatment. Moreover, sometimes subsequent visits that require examination and testing need to be done offline. Therefore, the two areas are divided into white and gray, indicating that they may be online or offline (Figure 1). Then, pharmacists check the electronic prescriptions, and drugs can be delivered by a third-party logistics enterprise cooperating with the hospital or can be picked up at a nearby pharmacy. Patients can also accept door-to-door diagnosis and treatment out of the hospital.

**Table 1:** The relationship and difference between others with internet hospitals.

Noun	Definition	Relationship and difference with internet hospitals
Online health	The use of internet technology to provide patients and the public with disease diagnosis, treatment programs, prescriptions, and other services [21].	It is one of the services provided by internet hospitals.
Mobile medicine	Emerging mobile communications and network technologies for health care systems [24]. It is via mobile and wireless devices, and the sharing of that information between patients and providers such as personal digital assistants, mobile phones, and satellite communications.	Internet hospitals have a variety of digital media, such as portals, public medical platforms, and apps.
Telemedicine	Use of telecommunications technologies to provide medical information and services [25].	Internet hospitals are a platform to provide technical support for remote diagnosis and treatment.

### Status Analysis of Internet Hospitals

**Figure 2:**
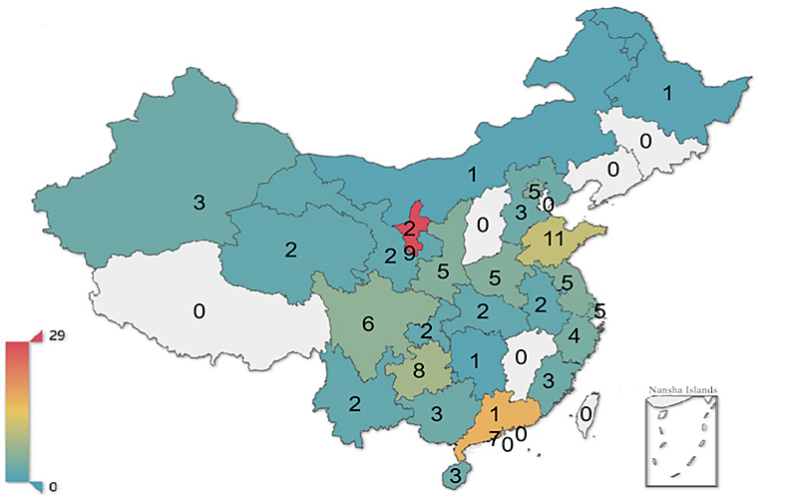
Heat map of number of internet hospitals in Chinese provinces.

As of January 2019, 25 of the 34 provinces or municipalities (73.5%) had registered a total of 130 internet hospitals in China, ranging from 0 to 29 per administrative region (Figure 2). There has been a growth trend from the establishment of internet hospitals up to the beginning of 2019. In the years from 2014 to 2018, 1 (0.8%), 5 (3.8%), 42 (32.3%), 65 (50.0%), and 17 (13.1%) hospitals were launched, respectively. The top 11 provinces and cities accounted for 100/130 (76.9%) internet hospitals. We can conclude that the number of internet hospitals grew very slowly between the end of 2017, when the establishment of internet hospitals was discouraged, and the end of 2018, when policies for comprehensive regulation were introduced.

Based on the general classification of internet hospital services and based on our data and the national policy documents, services provided by internet hospitals include four classifications, each referring to the service content for patients (Table 2). Convenience services help patients to operate online to simplify offline procedures and learn health knowledge independently. Online health means that patients can communicate with doctors to consult for health problems or obtain the diagnosis and prescriptions through the internet. Telemedicine is medical cooperation between institutions initiated by the hospital to other hospitals when patients seek medical treatment offline. Moreover, related industries refer to the content that needs to be provided by a third party, such as health records and drug-related enterprises.

**Figure 3:**
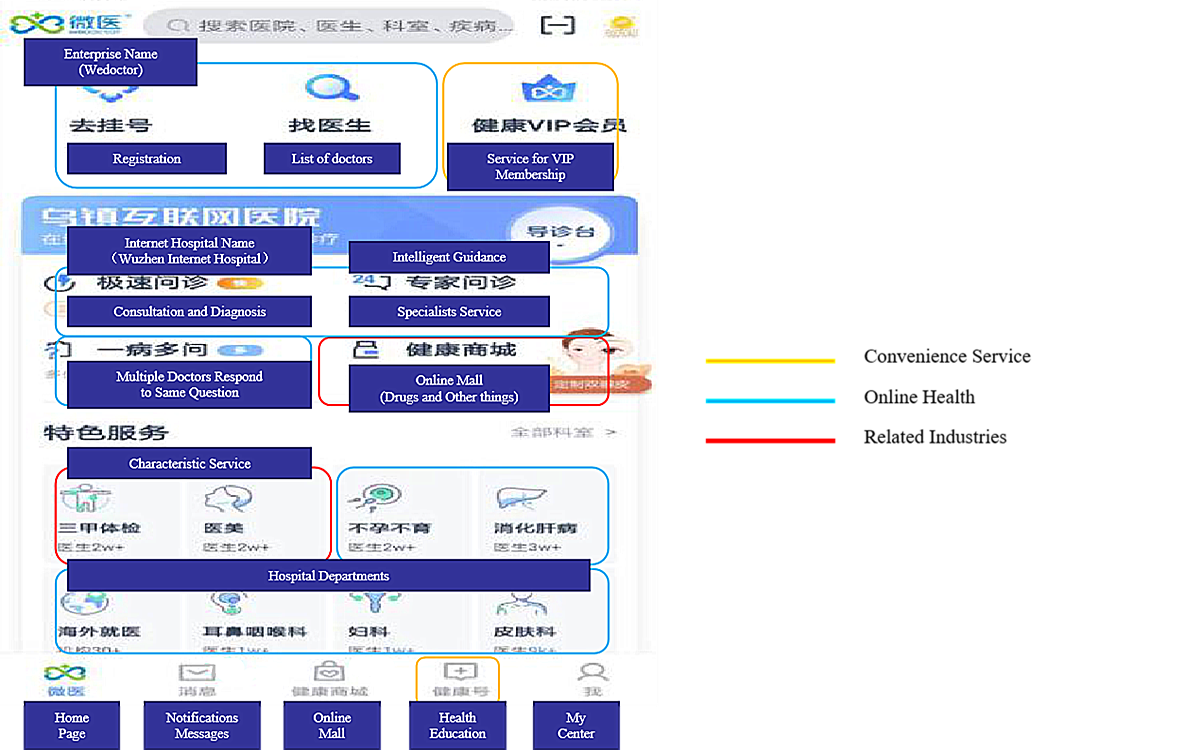
Example of internet hospital app interface.

We randomly selected the screenshot of the homepage of 1 Chinese internet hospitals app (Patient Client) (Figure 3); the content is based on 3 of the 4 classifications above except telemedicine between institutions.

**Table 2:** Internet hospital service classification.

Classification	Content
Convenience services	Intelligent guidance, registration, guidance, mobile payment, inspection report query, cost query [26], medical feedback, health education
Online health	Health consultation and follow-up for common and chronic diseases
Telemedicine (institution)	Providing on-line medical services initiated by institutions
Related industries	Drug order, drug distribution, health records

Every internet hospital contains specific content in the introduction, such as a booking service (online booking of a medical expert appointment within the medical facilities), but also health education (the hospital provides a wide range of medical knowledge about drugs, chronic disease knowledge lectures, information about national health policy, and so forth). Through internet portals, the patient can query to book appointments and can check reports. Doctors, via the outpatient internet service, can provide electronic prescription service to patients, including online prescriptions and online payment records. The network outpatient service for patients offers electronic prescription services, including online prescription, online payment dispensing, and others.

Based on our data, internet hospitals can be divided into three categories, and each has relatively successful typical cases. In the first category, the government is the initiator whose primary purpose is to achieve a unified standard of regional population health management, such as the Sichuan Province internet hospital, or the Zhejiang Province internet hospital. The second category is initiated by hospitals whose primary purpose is to expand the scope and intensity of hospital services so that people can continue to see their doctors. For example, the First Affiliated Hospital of Zhejiang University, a well-known tertiary hospital in China, opened their internet hospital to provide a platform for their doctors to provide services online. The third category is initiated by enterprises whose purpose is to connect patients with practicing doctors who, through these efforts, can increase the number of patients, and thereby, gain benefits. Also, they can improve the accessibility of health services for patients, and the increase in patients can improve doctors’ income and skills. This category includes some enterprises such as Haodf (Yinchuan Intelligent internet hospital), Ping An Good Doctor (Ping An Good Doctor internet hospital), Wedoctor (Wuzhen internet hospital). These were the first to utilize internet plus in the medical industry to carry out the sales of light drug consultations, health management, and other services. Even though these three categories can be separate, the effective delivery of medical services requires the cooperation of doctors, patients, hospitals, governments, and enterprises. Based on our database of internet hospitals, 38/130 (29.2%) were initiated by the government and hospital, and 92/130 (70.8%) were initiated by enterprises; we can see that each of them undertakes different roles from the perspective of stakeholders. The sources of doctors and patients are also different (Table 3).

**Table 3:** Relevant parties of internet hospital model.

Initiator	Integration of government services for guidance	Longitudinal hospital-led service	Horizontal enterprise-led service
Government	Establishment of internet medical platform at administrative level	The government is responsible for examination, approval and supervision	The government is responsible for examination, approval and supervision
Hospital	Establishment of online hospital districts in local medical institutions	Transfer offline services to online	Conditions and requirements on which services must provide and carry out entity-based
Enterprise	Contractor provides technical and medical resources support	Contractor provides technical support	Invest in construction for profit
Doctor	The contractor platform registered physicians and physicians from regional medical institutions provide services.	Doctors belonging to the hospital	Multipoint practicing doctor
Patient	Patients in administrative areas	Hospital service area mainly for patients, supplemented by network patients	Internet patients

## Discussion

### Principal Findings

The most striking finding in our study is the level of integration of ordinary hospital services with the internet hospital. While enterprises have initiated most internet hospitals (92/130, 70.8%), the dominant model is an integration of the internet platform with existing hospital and government services. The construction of the internet hospitals in China is a two-way force, to a certain extent, as a joint effort by the government and the market to alleviate the coexistence of a shortage of medical resources and wasted medical supplies. 

On the one hand, health resources are abundant in the east, while they are relatively few in the midwest regions [27]. In rural areas with scarce medical resources, the government and hospitals hope to optimize resources through telehealth, so that patients can contact other remote doctors to solve medical problems, and promote high-quality medical resources in the east to meet the needs of patients in the midwest; therefore, there are many internet hospitals in the central and western regions of China. The internet enables people to overcome geographic barriers to access health care services.

On the other hand, the e-commerce giants (such as Taobao or Jingdong) have been involved with the pharmaceutical industry, providing online drug shopping and health consulting for consumers, but they cannot directly sell prescription drugs [28]. The enterprises follow China’s internet medical regulation policies to create offline hospitals or cooperate with the existing medical institutions in building internet hospitals to ensure that they have implemented the whole process by the closed loop (doctor-patient-hospital medical services). With the rise of the industry, many regions with developed internet technologies took the initiative to enter the internet medical field and set up internet hospitals in developed cities to serve more patients.

### Internet Hospital Service Depends on the Integration of Online and Offline Services

The development of online medical services and health services in the new integrated model has several clear benefits. Patients can use the portal and medical information systems to improve communication with health care providers. They can take advantage of internet technology, reduce registration time, and reduce treatment times [29]. The richness of internet medical treatment can meet the diversified health needs of patients, to a certain extent, and also help to realize the continuity of medical services for patients [30,31].

Despite these advantages, it is also clear that internet hospitals cannot replace many of the core functions of physical hospitals. The internet hospital Management Measures stipulates two situations for the use of internet medical treatment in practice. In the first case, when a patient visits a physical medical institution and the institution initiates contact with other doctors to consult through an internet hospital, the physician can provide diagnostic advice and prescriptions. In the other case, patients do not consult with physical medical institutions. Doctors can then only offer follow-up to patients for common and chronic diseases through the internet hospital. The internet hospital includes many internet-based medical services, but it does not have many necessary medical resources and physical infrastructure online such as sickbeds, diagnostic equipment, and operating conditions for medical services. Therefore, the reality is that most internet hospital online services still only offer a supplement to the traditional nonclinical parts such as booking, registration, guidance, payment, and other convenience services [32].

Moreover, internet hospitals cannot provide the first diagnosis and complex disease follow-up after a diagnosis, so they cannot be involved in some of the core business of medical care, limiting the utilization rate [11,33]. For example, dermatology mainly relies on visual data to make a diagnosis. Usually, doctors with rich clinical experience can make a clear judgment after seeing the skin photos, while some departments rely more on examination and monitoring for diagnosis [34]. Meanwhile, in health consultation, doctors can conduct triage based on patient descriptions or video images, and doctors need to master additional characteristics of the patient’s integrated circumstance and ensure that they provide valid information for the medical records on the internet to provide online follow-up. Some medical services (first visit and severe illness) must be provided by offline hospitals, while others (nonclinical, health consultation, and follow-up diagnosis) can conveniently be realized online. Internet hospitals have stimulated the advantages of online and offline services to meet the medical needs of patients better and release high-quality medical resources (health workforce, equipment, etc) for offline hospitals [35].

### Different Initiators of Internet Hospitals May Have Different Development Directions

The government, hospitals, and enterprises are the initiators of the three ways of building internet hospitals, and they are also relevant stakeholders. The government is the policymaker, the main body of industry supervision, and plays an irreplaceable role [36]. The primary purpose of the government as the initiator is to integrate medical resource information and population health information in the region to form a database of population information, electronic health records, electronic medical records, and other data in the entire region [37,38]. As the foundation for the construction of internet hospitals, hospitals are providers of offline medical services. The primary purpose of an internet hospital with a physical hospital as the initiator is to extend the hospital's service process to the internet, extend the medical service capabilities of the physical hospital to the patient's home, and provide remote follow-up services for patients [39]. The enterprise is the technical support built by the internet hospital and maintains the regular operation of the platform. When enterprises are the main initiators of the internet hospital, their focus is on increasing operating income and achieving profitability. From the perspective of doctors and patients in internet hospitals, the source of doctors and patients for internet hospitals initiated by enterprises is significantly different from the source of doctors and patients when initiated by the government and hospitals. The doctors provided by internet hospitals built by the government and hospitals are qualified doctors within the administrative area, and the scope of their service is still local patients with a small number of cross-province patients. The service scope of the internet hospital built by enterprises is registered doctors and patients nationwide. Nevertheless, at least for the time being, enterprise-initiated internet hospitals still have to rely on existing physical infrastructure. If that should change and such hospitals can operate more independently, it would present a significant challenge for government regulation and supervision.

### Limitations

This study has several limitations. First, although this study attempts to describe the current situation of all internet hospitals in China, there may be missing items because data collection relied upon web-based search engines. Second, this research sample only included internet hospitals up to the beginning of 2019, which is the initial stage of the internet hospital industry (from its initiation and growth from very few to a substantial number), but it did not include the continuous replication of the internet hospital model in 2019. We will include these data in future studies to further analyze the relationship between the number of internet hospitals and the amount of services they provide. Third, this study did not investigate the actual online-offline conversion rate of internet hospitals to evaluate the service capacity of the hospitals. It is necessary to conduct research from the perspective of doctors, patients, and managers to further study stakeholders’ views on internet hospitals and to analyze the influencing factors of internet hospital usage in the current situation.

### Conclusions

Internet hospitals redefined the concept of medical service provision through telecommunications technology and began to revolutionize the health sector [40]. It is praiseworthy that China innovates the internet hospital model in the field of internet health care so that more people can receive medical resources to which they did not previously have access. The most significant value of the internet lies in the realization of the transfer of medical centers, from traditional hospitals as the main body to a more patient-oriented model where people can receive medical services and health care without leaving home. Currently, although the government has put forward management measures, they are still unable to achieve an internet hospital that provides the core functions of medical service. The government still needs to develop more suitable content to form a systematic internet medical model and to explore the regulatory scope to provide more development freedom for the internet hospital industry. It is essential to realize the central idea of the internet hospital—hospitals have more space to treat diseases, and patients save time [41].
